# Stimulation of protein synthesis by optogenetic and chemical induction of excitatory synaptic plasticity in hippocampal somatostatin interneurons

**DOI:** 10.1186/s13041-022-00967-y

**Published:** 2022-09-19

**Authors:** Ève Honoré, Inês Belo do Nascimento, Isabel Laplante, Jean-Claude Lacaille

**Affiliations:** Centre for Interdisciplinary Research on Brain and Learning, Research Group on Neural Signaling and Circuits, Department of Neurosciences, Université de Montréal, P.O. Box 6128, Station Downtown, QC H3C 3J7 Montreal, Canada

**Keywords:** GABA interneurons, Hebbian LTP, Late LTP, SUnSET assay, Puromycin translation assay, Raptor, Translation

## Abstract

Somatostatin-expressing interneurons (SOM-INs) are a major subpopulation of GABAergic cells in CA1 hippocampus that receive excitation from pyramidal cells (PCs) and provide feedback control of synaptic inputs onto PC dendrites. Excitatory synapses from PCs onto SOM-INs (PC-SOM synapses) exhibit long-term potentiation (LTP) mediated by type 1a metabotropic glutamate receptors (mGluR1a). LTP at PC-SOM synapses translates in lasting regulation of metaplasticity of entorhinal and CA3 synaptic inputs on PCs and contributes to hippocampus-dependent learning. A persistent form of PC-SOM synapse LTP lasting hours is prevented by blockers of transcription and translation, and a more transient form of PC-SOM synapse LTP lasting tens of minutes requires mTORC1-signaling, suggesting an involvement of protein synthesis. However, the role of protein synthesis in these forms of plasticity has not been directly demonstrated. Here we use the SUrface SEnsing of Translation (SUnSET) assay of protein synthesis to directly show that the induction protocols for both forms of LTP at PC-SOM synapses stimulate protein synthesis in SOM-INs. Moreover, protein synthesis stimulated by persistent LTP induction was prevented in mice with a SOM-IN conditional knock-out of Raptor, an essential component of mTORC1, indicating a critical role of mTORC1 in the control of translation in PC-SOM synapse plasticity. Moreover, protein synthesis induced by both forms of LTP may share common mechanisms as transient LTP induction occluded further stimulation of protein synthesis by persistent LTP induction. Our findings highlight a crucial role of protein synthesis and its control by mTORC1 in SOM-INs that is important for hippocampus-dependent memory function.

**Supplementary Information:**

The online version contains supplementary material available at 10.1186/s13041-022-00967-y.

## Introduction

Somatostatin-expressing interneurons in CA1 hippocampus (SOM-INs) are a major subpopulation of local inhibitory interneurons [1]. SOM-INs receive their major excitatory inputs from local pyramidal cells (PCs), and, in turn, provide feedback inhibition onto dendrites of PCs [1, 2]. SOM-INs regulate PC synaptic integration [3], action potential rate and burst firing [4] as well as synaptic plasticity [2, 5, 6], and are critical for contextual fear and spatial learning [6, 7, 8, 9]. A notable feature of SOM-INs is long-term plasticity of their excitatory synapses from PCs (PC-SOM synapses). These synapses show a transient form of Hebbian long-term potentiation (LTP) mediated by type 1a metabotropic glutamate receptors (mGluR1a) that lasts tens of minutes [2, 9, 10, 11]. Importantly, optogenetic induction of transient LTP at PC-SOM synapses involves mechanistic target of rapamycin complex 1 (mTORC1) signaling and regulates hippocampal memory function [9]. In addition, contextual fear learning induces a persistent mGluR1a-dependent LTP at PC-SOM synapses, that lasts 24 h and requires mTORC1 signaling [6]. Repeated mGluR1 chemical stimulation or electrical theta burst stimulation (TBS) in slices also elicit persistent LTP at PC-SOM synapses which lasts hours and is transcription- and translation-dependent [6, 12]. Moreover, down-regulation of mTORC1 selectively in SOM-INs impairs mGluR1a-dependent persistent LTP and contextual fear and spatial memory consolidation, suggesting a necessary role of PC-SOM synapse LTP in hippocampal memory [6]. Conditional knock-in of the non-phosphorylatable translation initiation factor eIF2α (eIF2α^S51A^) in SOM-INs upregulates their general mRNA translation, gates CA1 network plasticity and increases long-term contextual fear memory [8]. Thus, protein synthesis and both transient and persistent long-term synaptic plasticity in SOM-INs are critically implicated in hippocampal learning and memory [6, 8, 9]. However, a direct link remains to be demonstrated between transient and persistent LTP at PC-SOM synapses and protein synthesis in SOM-INs via mGluR1a and mTORC1.

Here, we investigate the involvement of protein synthesis in plasticity at PC-SOM synapses using the SUrface SEnsing of Translation (SUnSET) assay [13] in SOM-INs in combination with chemical and optogenetic induction of persistent and transient forms of LTP, respectively, in slices from transgenic mice expressing EYFP in SOM-INs. We test also the role of mTORC1 in protein synthesis, using mice with a conditional knockout of the essential component of mTORC1, regulatory-associated protein of mTOR (Raptor), in SOM-INs.

## Results

First, we established the specificity of puromycin labeling in the SUnSET assay [8, 13] of EYFP-expressing CA1 SOM-INs of acute hippocampal slices obtained from *Sst*^ires−Cre^;*Rosa26*^lsl−EYFP^ mice (SOM-EYFP-WT mice) [6] (Additional file 1—Materials and methods). Puromycin immunolabeling was present in approximately 25% of EYFP-expressing SOM-INs, but was absent in slices not treated with puromycin, or slices processed for SUnSET assay but without puromycin primary antibody (Additional file 2: Fig. S1). These results confirm the specificity of puromycin immunolabeling and indicate a detectable basal level of protein synthesis in SOM-INs of hippocampal slices.

**Fig. 1:**
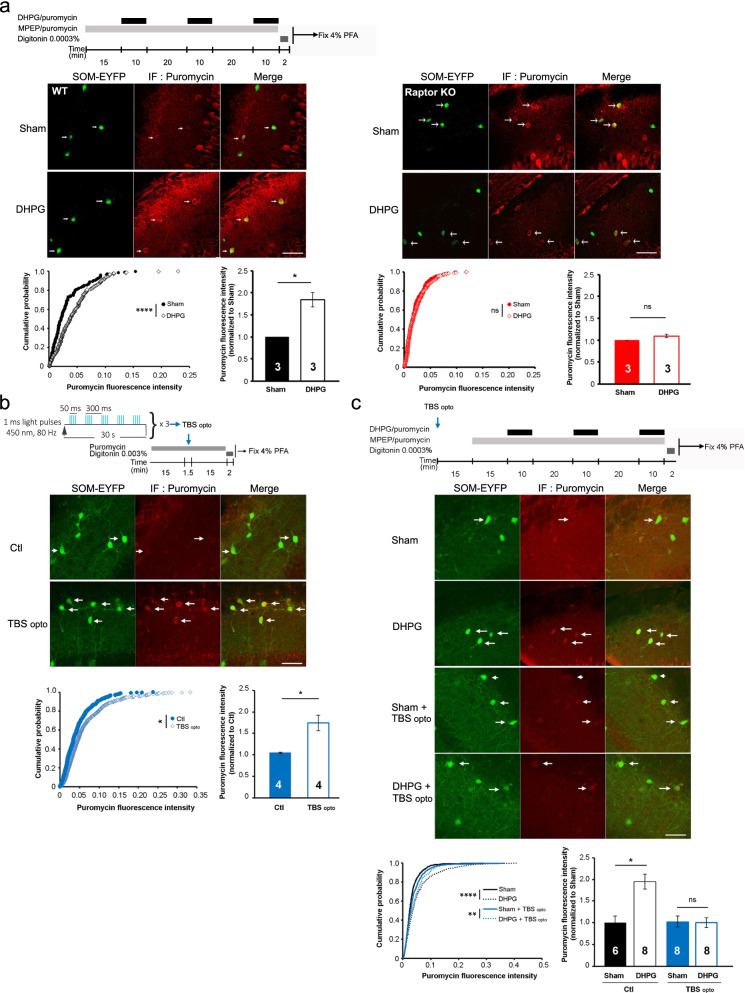
Stimulation of protein synthesis in SOM-INs by chemical and optogenetic induction of persistent and transient LTP, respectively. **a** Protocol of chemical persistent LTP induction (top left), representative images (middle), cumulative distribution plots and summary bar graphs (bottom; each group 3 independent slice experiments from 3 animals, 3–6 sections analyzed per experiment) of puromycin immunofluorescence in SOM-INs of control SOM-EYFP-WT mice (left) and conditional SOM-EYFP-Raptor-KO mice (right), showing increase in fluorescence after DHPG treatment relative to Sham-treatment in control mice, but not in conditional knockout mice. Summary bar graph (mean ± SEM; SOM-EYFP-WT mice, Sham = 395 cells and DHPG = 396 cells; SOM-EYFP-Raptor KO mice, Sham = 457 cells and DHPG = 487 cells). **b** Protocol of optogenetic (TBS_opto_) transient LTP induction (top), representative images (middle), cumulative distribution plot and summary bar graph (bottom; each group 4 independent slice experiments from 2 animals, 4–6 sections analyzed per experiment) of puromycin immunofluorescence in SOM-INs of SOM-EYFP-WT mice, showing an increase in fluorescence after TBS_opto_ relative to control (unstimulated) slices. Summary bar graph (mean ± SEM; Control 570 cells and TBS_opto_ 761 cells). **c** Protocol of consecutive induction of TBS_opto_ (in absence of puromycin) and repeated mGluR1 (in presence of puromycin) LTP (top), representative images (middle), cumulative distribution plot and summary bar graph (bottom; each group 6–8 independent slice experiments from 3–4 animals, 3–6 sections analyzed per experiment) of puromycin immunofluorescence in SOM-INs of SOM-EYFP-WT mice, showing that the increase in fluorescence after DHPG treatment is impaired by prior application of TBS_opto_. Summary bar graph (mean ± SEM; Ctl, Sham 781 cells and DHPG 805 cells; TBS_opto_, Sham 925 cells and DHPG: 869 cells). In all panels: arrows indicate cells with colocalization of EYFP with puromycin immunofluorescence; scale bars, 50 µm; Kolmogorov–Smirnov tests (cumulative distribution tests) or Student’s *t*-tests (group mean tests), * p < 0.05, ** p < 0.01, **** p < 0.0001 and ns not significant

Synaptic mechanisms that induce chemical late LTP in CA1 pyramidal cells (NMDAR, cAMP) [14] are different from the induction mechanisms implicated in chemical persistent LTP in CA1 SOM interneurons (mGluR1a) [6, 12]. So next, we examined if repeated mGluR1 chemical stimulation, an effective protocol for induction of mTORC1-mediated persistent LTP at PC-SOM synapses [6, 12], increases protein synthesis in SOM-INs of control SOM-EYFP-WT mice. After repeated application of the mGluR1/5 agonist (S)-3,5-dihydroxyphenylglycine (DHPG) in presence of the mGluR5 antagonist 2-methyl-6-(phenylethynyl)-pyridine (MPEP), puromycin immunolabeling was increased in SOM-INs relative to sham-treated slices (Fig. 1a), indicating that chemical induction of persistent LTP stimulates protein synthesis in SOM-INs. We tested the role of mTORC1 in persistent LTP-induced protein synthesis using mice with a conditional knockout of Raptor, an essential component of mTORC1, in SOM-INs (*Sst*^ires−Cre^;*Rosa26*^lsl−EYFP^;*Rptor*^fl/fl^ knock-out mice; SOM-EYFP-Raptor-KO mice) [6]. Repeated mGluR1 stimulation of slices from SOM-EYFP-Raptor-KO mice failed to increase puromycin immunolabeling in SOM-INs (Fig. 1a), indicating that protein synthesis elicited by induction of persistent LTP in SOM-INs is mediated by mTORC1 signaling. Basal level of puromycin immunofluorescence in SOM-INs of sham-treated slices was similar in SOM-EYFP-WT and SOM-EYFP-Raptor-KO mice (Additional file 2: Fig. S2a), suggesting undetectable mTORC1 control of basal protein synthesis in SOM-INs. We also examined puromycin immunofluorescence in CA1 stratum pyramidale after repeated mGluR1 stimulation. DHPG treatment did not increase stratum pyramidale puromycin immunofluorescence in slices of SOM-EYFP-WT or SOM-EYFP-Raptor-KO mice (Additional file 2: Fig. S2b-c), suggesting that induction protocol for persistent LTP at PC-SOM synapses stimulates protein synthesis in SOM-INs, but may not in PCs.

Next, we determined if optogenetic theta-burst stimulation (TBS_opto_), an effective protocol to induce transient LTP at PC-SOM synapses [6, 9], increases protein synthesis in SOM-INs. hChR2 was expressed in PCs by CA1 injections of AAV2/9-CaMKIIa-hChR2(E123T/T159C)-mCherry in SOM-EYFP-WT mice, as previously [9] (Additional file 1—Materials and methods). Optogenetic theta burst stimulation (TBS_opto_) was then given as previously [9] in slices prepared for SUnSET assay (Fig. 1b). Puromycin immunolabeling was increased in SOM-INs after TBS_opto_ relative to unstimulated slices from hChR2-expressing mice (Fig. 1b), indicating that optogenetic induction of transient LTP stimulates protein synthesis in SOM-INs.

Next, we examined if protein synthesis elicited in SOM-INs by TBS_opto_ and repeated mGluR1 stimulation interact with each other. TBS_opto_ was given to slices in absence of puromycin and repeated mGluR1 stimulation was applied 30 min later in presence of puromycin (Fig. 1c). In control slices (without prior TBS_opto_), puromycin immunofluorescence was increased in SOM-INs after DHPG treatment (Fig. 1c). But in slices with prior TBS_opto_, the increase in puromycin immunofluorescence after DHPG treatment was impaired (Fig. 1c). Thus, induction of transient LTP prevents further stimulation of protein synthesis by persistent LTP induction, suggesting both forms of LTP share signaling mechanisms.

## Discussion

Here, we demonstrate that protein synthesis is stimulated in SOM-INs by induction protocols for transient and persistent LTP at PC-SOM synapses. Moreover, stimulation of protein synthesis by persistent LTP induction requires mTORC1 signaling. Transient LTP induced by TBS_opto_ is known to be mTORC1-dependent [9], thus protein synthesis induced by TBS_opto_ is also likely mTORC1-mediated. Our results suggest a causal role of protein synthesis in PC-SOM LTP because both transient LTP [9] and persistent LTP [6] were previously shown to be blocked in mice with conditional knockout of *Rptor* in SOM-INs. Since transient and persistent LTP at PC-SOM synapses contribute to long-term contextual fear and spatial learning [6, 9], our findings highlight a crucial role of translational control by mTORC1 in SOM-INs that is important for hippocampus-dependent memory processes.

Protein synthesis induced by optogenetic and repeated mGluR1 stimulation share mechanisms since TBS_opto_ induction occludes activation of protein synthesis by subsequent repeated mGluR1 stimulation. These results are consistent with previous evidence that TBS_opto_ induction in vivo prior to contextual fear conditioning impairs learning-induced potentiation of PC-SOM synapses, and reduces contextual fear memory consolidation [9]. Prior induction of persistent LTP by repeated mGluR1 stimulation in SOM-INs was also found to occlude subsequent transient LTP elicited by electrical TBS [12]. Our findings indicate that the interaction may occur upstream of protein synthesis. Thus, it will be of interest to determine where the occlusion occurs in the signaling cascade between mGluR activation and protein synthesis.

Our finding of long-term potentiation of PC-SOM synapses mediated by mGluR1a and associated with protein synthesis in SOM-INs, contrasts with the long-term depression (LTD) of Schaffer collateral synapses in PCs mediated by mGluR5 and protein synthesis [15]. Our observation that stimulation of protein synthesis by mGluR1-mediated persistent LTP induction protocol was observed in SOM-INs and absent in stratum pyramidale, is consistent with the requirement of activation of mGluR5 for LTD and protein synthesis in PCs [15]. Given the functional specificity of mGluR stimulated protein synthesis in SOM-INs (potentiation of synapses) and PCs (depression of synapses), it will be important to identify the cell-specific mRNAs controlled by synaptic activity and mTORC1 which determine the type of synaptic plasticity in SOM-INs and PCs.

## Supplementary Information

**Additional file 1.** Materials and methods.

**Additional file 2: Figure S1.** Specificity of puromycin labeling in SUnSET assay. (a) Diagram of puromycin entering the ribosomal A-site, arrest of protein synthesis and release of premature peptides, later detected using a puromycin specific antibody. (b) Representative images of EYFP and puromycin immunofluorescence showing specificity of puromycin antibody in absence (upper panels) or presence (middle panels) of puromycin, and no puromycin labeling without puromycin antibody (bottom panels). Hippocampal slices were exposed with or without puromycin to Sham-treatment of late LTP protocol. Arrows indicate cells with colocalization of EYFP and puromycin fluorescence signal. Scale bar, 100 µm. (c) Summary bar graph of puromycin colocalization in EYFP cells expressed as percentage of total EYFP cells (4 independent experiments with 1-2 sections analyzed per experiment, in each group; n = 275 EYFP cells with puromycin co-localization). **Figure S2.** Intact SOM-IN basal protein synthesis in mice with conditional *Rptor* knock-out, and unchanged pyramidal cell layer puromycin immunofluorescence after chemical persistent LTP induction. (a) Representative images and summary bar graph (each group 5 independent slice experiments from 5 animals, 1-5 sections analyzed per experiment) of puromycin immunofluorescence in SOM-INs of SOM-EYFP WT and SOM-EYFP-Raptor KO mice, showing no difference of puromycin fluorescence in SOM-INs (sham-treatment) of control and knockout mice. Arrows indicate cells with colocalization of EYFP with puromycin immunofluorescence. Summary bar graph (mean ± SEM; WT 570 cells and Raptor KO 722 cells). (b-c) Representative images and summary bar graphs (each group 3 independent slice experiments from 3 animals, 1-3 sections analyzed per experiment) of puromycin immunofluorescence in the CA1 pyramidal layer of SOM-EYFP WT (b) and SOM-EYFP-Raptor KO (c), showing no difference in puromycin fluorescence following chemical persistent LTP induction. Summary bar graph (mean ± SEM; number of fields of view for WT, Sham 53 and DHPG 88; for Raptor-KO, Sham 75 and DHPG 101). Scale bars, 50 µm. Student’s *t*-tests, ns not significant

## Data Availability

All data and materials are available upon request.

## Abbreviations

DHPG: (S)-3,5-dihydroxyphenylglycine
EYFP: Enhanced yellow fluorescent protein
LTD: Long-term depression
LTP: Long-term potentiation
mGluR1a: Metabotropic glutamate receptors type 1a
MPEP: 2-Methyl-6-(phenylethynyl)-pyridine
mTORC1: Mechanistic target of rapamycin complex 1
PC: Pyramidal cell
SOM: Somatostatin
SOM-EYFP-Raptor KO: *Sst*^Ires^^−^^Cre^;*Rosa26*^lsl^^−^^EYFP^;*Rptor*^fl/^^fl^ knock-out mice
SOM-EYFP-WT: *Sst*^Ires^^−^^Cre^;*Rosa26*^lsl^^−^^EYFP^ mice
SOM-IN: Somatostatin-expressing interneuron
SUnSET: SUrface SEnsing of Translation
TBS: Theta burst stimulation
TBS_opto_: Optogenetic theta burst stimulation
