# Supplementary material for: Stimulation of protein synthesis by optogenetic and chemical induction of excitatory synaptic plasticity in hippocampal somatostatin interneurons
Source: Mol Brain. 2022 Sep 19;15:81. doi: 10.1186/s13041-022-00967-y (PMC9484204; doi:10.1186/s13041-022-00967-y)
Supplement: Supplementary file 1 — Additional file 1. Materials and methods. [file 13041_2022_967_MOESM1_ESM.docx]

**Materials and methods**

*Animals*

All animal procedures and experimental protocols were performed in accordance with the guidelines of the Animal Care Committee of University of Montreal and of the Canadian Council on Animal Care.

*Transgenic mice lines*

Knock-in mice expressing Cre under a ribosomal entry site (IRES) downstream of *Sst* locus (*Sst*^ires-Cre^ mice; The Jackson Laboratory, Bar Harbor, ME; JAX #013044) (1) were crossed with *Rosa26*^lsl-EYFP^ reporter mice (Ai3; JAX#007903) (2) to generate mice with Cre-dependent Enhanced Yellow Fluorescent Protein (EYFP) expression in SOM-INs (SOM-EYFP-WT mice) (3). *Sst*^ires-Cre^;*Rosa26*^lsl-EYFP^ mice were crossed with floxed *Rptor* mice (JAX#013188) (4) for cell-specific knock-out of *Rptor* in SOM cells (SOM-EYFP-Raptor-KO mice) (3). Heterozygous offsprings were backcrossed together and homozygous animals were isolated. Mice were housed 2–5 animals per cage and given *ad libitum* access to food and water, in temperature (~22°C) and humidity (~55%) controlled rooms with a normal 12h light/dark cycle. Experiments were carried out on 4-9 weeks old mice from both sexes.

*Viral constructs injection*

Four to six weeks old mice were anesthetized with an intraperitoneal injection of ketamine (50 mg/kg) and xylazine (5 mg/kg) and placed in a stereotaxic apparatus (Stoelting). AAV2/9-CaMKIIa-hChR2(E123T/T159C)-mCherry (1.5x10^12^ particles/ml) was injected bilaterally in dorsal CA1 hippocampus (coordinates from bregma: -2.46 mm AP; ± 1.75 mm ML; -1.5 mm DV). Viral solution (0.8 µl) was delivered at a flow rate of 100 nl/min using a 10 µl Hamilton syringe coupled to a G26 beveled needle. The needle was left in place for at least 5 minutes after injection. Mice were allowed to recover for 2 weeks prior to slice experiments.

*Hippocampal slice preparation*

Mice were anesthetized with isoflurane inhalation and the brain was rapidly removed and placed in ice-cold sucrose-based cutting solution containing (in mM): 75 sucrose, 87 NaCl, 2.5 KCl, 1.25 NaH_2_PO_4_, 7 MgCl_2_, 0.5 CaCl_2_, 25 NaHCO_3_, 25 D-glucose (pH 7.4 and 330 mOsmol/L). Three hundred microns thick transverse dorsal hippocampal slices were cut with a Leica VT1000S vibratome (Leica, Germany). Slices were transferred for a recovery period of one hour in artificial cerebrospinal fluid (ACSF) at room temperature (20 –22°C) containing (in mM): 124 NaCl, 2.5 KCl, 1.25 NaH_2_PO_4_, 2 MgCl_2_, 2 CaCl_2_, 26 NaHCO_3_, 10 D-glucose, 1.3 ascorbic acid (pH 7.4 and 295-305 mOsmol/L). Both cutting solution and ACSF were saturated with 95% O_2_/5% CO_2_.

*Protein synthesis assay*

To measure protein synthesis in SOM-INs we used the immunohistochemical version of the assay called SUrface SEnsing of Translation (SUnSET) (also called puromycin translation assay) (5-8). Individual slices were pre-incubated for 15 minutes in ACSF containing puromycin (100 µM; Sigma-Aldrich, P8833) and saturated with 95% O_2_/5% CO_2_ at 30-33ºC. Slices received the LTP induction protocol (see below) in the presence of puromycin, followed by a post-incubation period of 2 minutes in digitonin (0.0003%; Sigma-Aldrich; D141). Slices were then fixed in 4% paraformaldehyde in 0.1 M phosphate buffer (PB) overnight at 4ºC before processing for immunofluorescence. Slices were rinsed in 0.01 M PB saline (PBS), cryoprotected with 30% sucrose in 0.1 M PB, and re-sectioned (50 µm thickness) using a Leica freezing microtome (SM 2000R). Sections were permeabilized in 0.3% Triton X-100 in 0.01 M PBS for 15 min. To block unspecific binding sections were incubated in 10% normal goat serum, 0.1% Triton X-100 in PBS for 1 hour, followed by overnight incubation at 4ºC with a mouse monoclonal IgG2a puromycin antibody (1/1000; Millipore #MABE343). Sections were then incubated at room temperature (1h30) with a goat anti-mouse IgG2a-conjugated to Rhodamine-Red™X (1/200) or Alexa Fluor™ 647 (1/200; for AAV2/9-CaMKIIa-hChR2(E123T/T159C)-mCherry experiments) and mounted in Prolong Diamond. Images were acquired with a confocal microscope (LSM510 or LSM880, Zeiss) at excitation wavelength 488 nm and 543 nm (Rhodamine-Red labeling) or 633 nm (Alexa Fluor 647 labeling). Images were acquired using the exact same parameters set on control sections. In each section, the intensity of puromycin immunofluorescence was quantified in EYFP-expressing SOM-INs in similar sized fields of view (150 x 150 µm) in *oriens-alveus* region of the CA1 hippocampus using ImageJ software (National Institute of Health; <https://imagej.nih.gov/ij/download.html>) by comparing integrated intensity in cells corrected for background fluorescence. In each immunofluorescence independent experiment, control and treated slices were processed together and analyzed using the same equipment settings. Immunofluorescence intensity of cells from treated slices was normalized to values of cells in associated control slices. In summary bar graphs, immunofluorescence intensity was expressed as mean of independent experiments (numbers for different experiments indicated in Figure 1 legend). For measurement of puromycin immunofluorescence in the CA1 pyramidal layer, the integrated fluorescence intensity was measured in similar sized fields of view (30 x 20 µm) in the pyramidal cell layer and corrected for background fluorescence.

*LTP induction protocols*

The protocol for chemical induction of persistent mGluR1-mediated LTP was as previously (9). Briefly, induction consisted of three applications (10 min duration each at 30 min intervals) of the mGluR1/5 agonist (S)-3,5-dihydroxyphenylglycine (DHPG, 5 µM, Abcam) in the presence of the mGluR5 antagonist 2-methyl-6-(phenylethynyl)-pyridine (MPEP, 40 µM, Abcam) and puromycin. Puromycin incubation was terminated after the last DHPG application.

The protocol for optogenetic theta burst stimulation (TBS_opto_) induction of transient LTP was as previously (10) in slices preincubated in ACSF and puromycin for 15 min, as described above. Briefly, whole field optogenetic stimulation of slices were elicited using a Quadruple Laser Diode Fiber Light Source (LDFLS_405/100_450/070_520/060_638/080; Doric Lenses Inc) coupled to Mono Fiberoptic Patchcords (MFP_200/240/900–0.22_2m_FC-ZF1.25(F), 200 mm Core diameter, 0.22 NA; Doric Lenses Inc) and hand-made fiber optic cannulas (optic fiber: FT200EMT, 200 mm Core diameter, 0.22 NA; ceramic ferrule: CFLC230-10; Thorlabs) positioned above the CA1 stratum oriens region of the slice with brief light pulses (450 nm; duration 1 ms). Optogenetic stimulation of CA1 pyramidal cells (TBS_opto_) consisted of 3 episodes (given at 30 s intervals) of 5 bursts (at 300 ms inter-burst interval) of 4 light pulses at 80 Hz. Slices were incubated in ACSF with puromycin for a further 15 min after the TBS_opto_ induction protocol. In experiments with TBS_opto_ preceding chemical LTP induction, slices received TBS_opto_ (or no light) without puromycin and, after 15 min, were exposed to the chemical LTP induction protocol in the presence of puromycin as detailed above.

*Statistical Analysis*

No statistical methods were used to predetermine sample size but our sample sizes are comparable to those used generally in the field. Statistical analysis was performed using Sigma Stat, Excel and ClampFit. Student’s *t* tests were used to determine difference between control and treatment groups, and Fisher tests to validate homoscedasticity. Kolmogorov-Smirnov tests were used to compare cumulative probability distributions. All data in the Figures are presented as mean ± SEM. Asterisks in Figures denote statistical significance levels for specified tests (* *p* < 0.05; ** *p* <0.01; *** *p* < 0.001; ns, not significant).

**References**

1. Taniguchi H, He M, Wu P, Kim S, Paik R, Sugino K, et al. A resource of Cre driver lines for genetic targeting of GABAergic neurons in cerebral cortex. Neuron. 2011;71(6):995-1013.

2. Madisen L, Zwingman TA, Sunkin SM, Oh SW, Zariwala HA, Gu H, et al. A robust and high-throughput Cre reporting and characterization system for the whole mouse brain. Nature neuroscience. 2010;13(1):133-40.

3. Artinian J, Jordan A, Khlaifia A, Honore E, La Fontaine A, Racine AS, et al. Regulation of Hippocampal Memory by mTORC1 in Somatostatin Interneurons. J Neurosci. 2019;39(43):8439-56.

4. Sengupta S, Peterson TR, Laplante M, Oh S, Sabatini DM. mTORC1 controls fasting-induced ketogenesis and its modulation by ageing. Nature. 2010;468(7327):1100-4.

5. Schmidt EK, Clavarino G, Ceppi M, Pierre P. SUnSET, a nonradioactive method to monitor protein synthesis. Nat Methods. 2009;6(4):275-7.

6. Goodman CA, Mabrey DM, Frey JW, Miu MH, Schmidt EK, Pierre P, et al. Novel insights into the regulation of skeletal muscle protein synthesis as revealed by a new nonradioactive in vivo technique. FASEB J. 2011;25(3):1028-39.

7. Ma T, Trinh MA, Wexler AJ, Bourbon C, Gatti E, Pierre P, et al. Suppression of eIF2alpha kinases alleviates Alzheimer's disease-related plasticity and memory deficits. Nature neuroscience. 2013;16(9):1299-305.

8. Sharma V, Sood R, Khlaifia A, Eslamizade MJ, Hung TY, Lou D, et al. eIF2alpha controls memory consolidation via excitatory and somatostatin neurons. Nature. 2020;586(7829):412-6.

9. Ran I, Laplante I, Bourgeois C, Pepin J, Lacaille P, Costa-Mattioli M, et al. Persistent transcription- and translation-dependent long-term potentiation induced by mGluR1 in hippocampal interneurons. J Neurosci. 2009;29(17):5605-15.

10. Asgarihafshejani A, Honore E, Michon FX, Laplante I, Lacaille JC. Long-term potentiation at pyramidal cell to somatostatin interneuron synapses controls hippocampal network plasticity and memory. iScience. 2022;25(5):104259.
