# Supplementary material for: Stimulation of protein synthesis by optogenetic and chemical induction of excitatory synaptic plasticity in hippocampal somatostatin interneurons
Source: Mol Brain. 2022 Sep 19;15:81. doi: 10.1186/s13041-022-00967-y (PMC9484204; doi:10.1186/s13041-022-00967-y)
Supplement: Supplementary file 2 — Additional file 2: Figure S1. Specificity of puromycin labeling in SUnSET assay. (a) Diagram of puromycin entering the ribosomal A-site, arrest of protein synthesis and release of premature peptides, later detected using a puromycin specific antibody. (b) Representative images of EYFP and puromycin immunofluorescence showing specificity of puromycin antibody in absence (upper panels) or presence (middle panels) of puromycin, and no puromycin labeling without puromycin antibody (bottom panels). Hippocampal slices were exposed with or without puromycin to Sham-treatment of late LTP protocol. Arrows indicate cells with colocalization of EYFP and puromycin fluorescence signal. Scale bar, 100 µm. (c) Summary bar graph of puromycin colocalization in EYFP cells expressed as percentage of total EYFP cells (4 independent experiments with 1-2 sections analyzed per experiment, in each group; n = 275 EYFP cells with puromycin co-localization). Figure S2. Intact SOM-IN basal protein synthesis in mice with conditional Rptor knock-out, and unchanged pyramidal cell layer puromycin immunofluorescence after chemical persistent LTP induction. (a) Representative images and summary bar graph (each group 5 independent slice experiments from 5 animals, 1-5 sections analyzed per experiment) of puromycin immunofluorescence in SOM-INs of SOM-EYFP WT and SOM-EYFP-Raptor KO mice, showing no difference of puromycin fluorescence in SOM-INs (sham-treatment) of control and knockout mice. Arrows indicate cells with colocalization of EYFP with puromycin immunofluorescence. Summary bar graph (mean ± SEM; WT 570 cells and Raptor KO 722 cells). (b-c) Representative images and summary bar graphs (each group 3 independent slice experiments from 3 animals, 1-3 sections analyzed per experiment) of puromycin immunofluorescence in the CA1 pyramidal layer of SOM-EYFP WT (b) and SOM-EYFP-Raptor KO (c), showing no difference in puromycin fluorescence following chemical persistent LTP induction. Summary bar grap [file 13041_2022_967_MOESM2_ESM.docx]

**
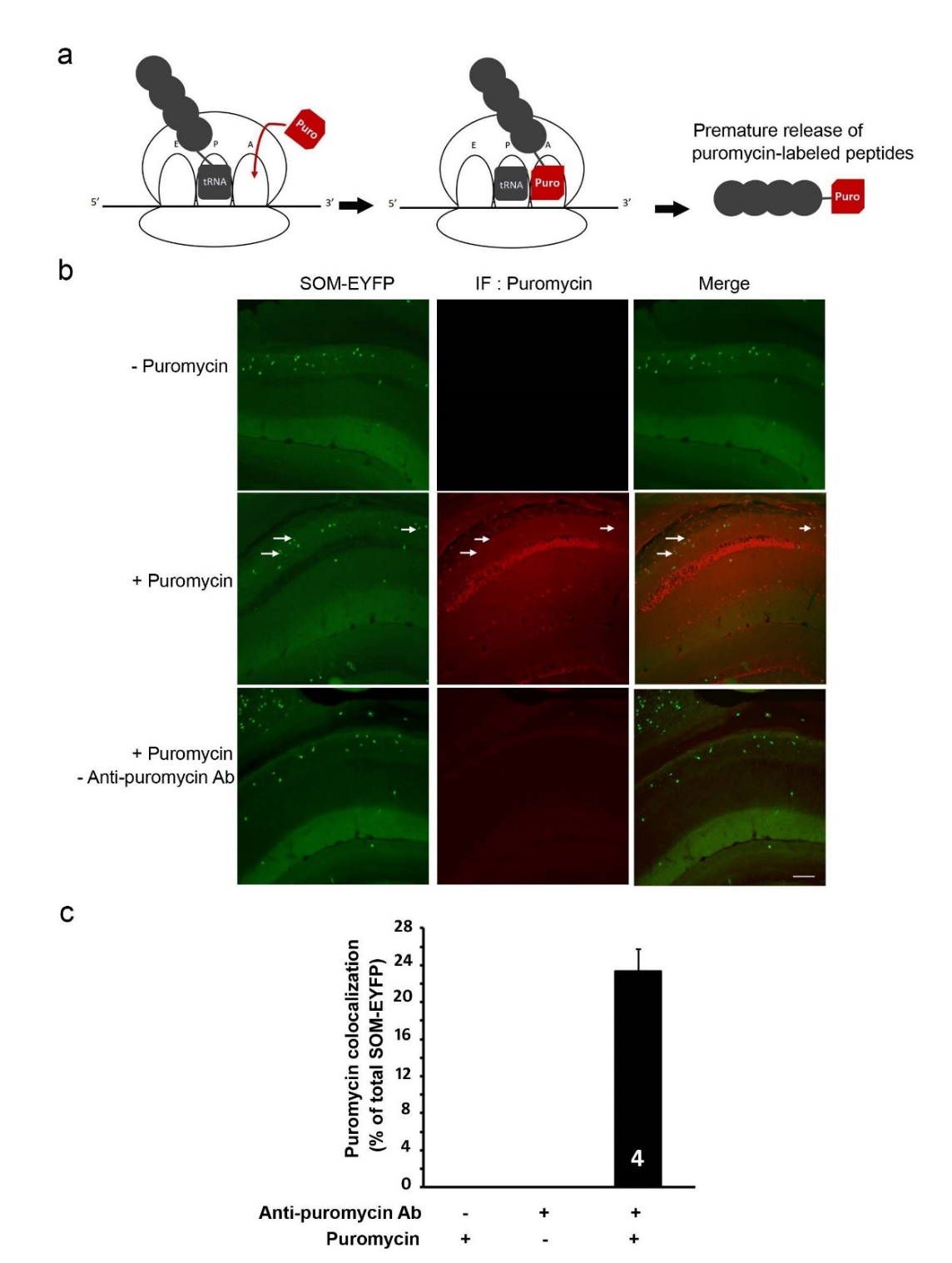
**

**Figure S1.** Specificity of puromycin labeling in SUnSET assay. (**a**) Diagram of puromycin entering the ribosomal A-site, arrest of protein synthesis and release of premature peptides, later detected using a puromycin specific antibody. (**b**) Representative images of EYFP and puromycin immunofluorescence showing specificity of puromycin antibody in absence (upper panels) or presence (middle panels) of puromycin, and no puromycin labeling without puromycin antibody (bottom panels). Hippocampal slices were exposed with or without puromycin to Sham-treatment of late LTP protocol. Arrows indicate cells with colocalization of EYFP and puromycin fluorescence signal. Scale bar, 100 µm. (**c**) Summary bar graph of puromycin colocalization in EYFP cells expressed as percentage of total EYFP cells (4 independent experiments with 1-2 sections analyzed per experiment, in each group; n = 275 EYFP cells with puromycin co-localization).

**
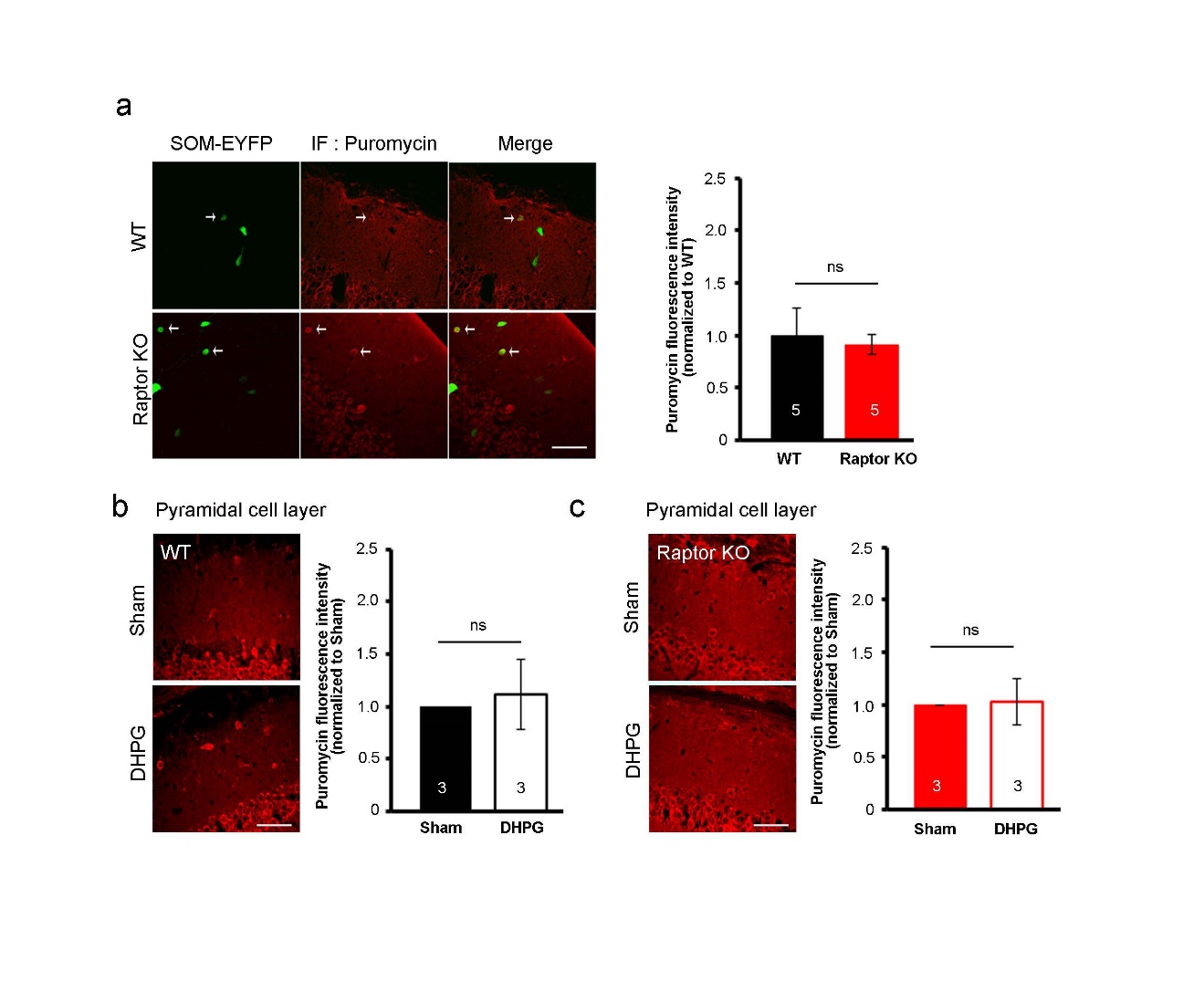
**

**Figure S2.** Intact SOM-IN basal protein synthesis in mice with conditional *Rptor* knock-out, and unchanged pyramidal cell layer puromycin immunofluorescence after chemical persistent LTP induction. (**a**) Representative images and summary bar graph (each group 5 independent slice experiments from 5 animals, 1-5 sections analyzed per experiment) of puromycin immunofluorescence in SOM-INs of SOM-EYFP WT and SOM-EYFP-Raptor KO mice, showing no difference of puromycin fluorescence in SOM-INs (sham-treatment) of control and knockout mice. Arrows indicate cells with colocalization of EYFP with puromycin immunofluorescence. Summary bar graph (mean ± SEM; WT 570 cells and Raptor KO 722 cells). (**b-c**) Representative images and summary bar graphs (each group 3 independent slice experiments from 3 animals, 1-3 sections analyzed per experiment) of puromycin immunofluorescence in the CA1 pyramidal layer of SOM-EYFP WT (**b**) and SOM-EYFP-Raptor KO (**c**), showing no difference in puromycin fluorescence following chemical persistent LTP induction. Summary bar graph (mean ± SEM; number of fields of view for WT, Sham 53 and DHPG 88; for Raptor-KO, Sham 75 and DHPG 101). Scale bars, 50 µm. Student’s *t*-tests, ns not significant.
